# The enhancement of the employer branding strategies of Polish hospitals through the detection of features which determine employer attractiveness: a multidimensional perspective

**DOI:** 10.1186/s12960-021-00620-0

**Published:** 2021-06-28

**Authors:** Beata Buchelt, Bernard Ziębicki, Joanna Jończyk, Joanna Dzieńdziora

**Affiliations:** 1 Department of Human Capital Management, Cracow University of Economics, Krakow, Poland; 2 Department of Organization and Management Methods, Cracow University of Economics, Krakow, Poland; 3 Faculty of Engineering Management, Bialystok University of Technology, Białystok, Poland; 4 Department of Management, WSB University, Dąbrowa Górnicza, Poland

**Keywords:** HRM, Employer branding (EB), Physicians, Nurses, Hybrid managers

## Abstract

**Background:**

Polish healthcare providers already struggle with a deficiency concerning human resources, especially with regard to doctors and nurses. Because of this, effective HRM interventions should be taken in order to attract and retain medical personnel. Employer branding is one such intervention because it not only results in improving the organization's reputation as an employer but also improving HRM practices. However, to create an effective employer branding strategy, a contextual approach should be taken. Because of this, the aim of the study is to assess the importance of various factors influencing medical personnel’s perception of a hospital’s attractiveness as an employer.

**Methods:**

The study was performed among 285 hospitals in Poland assuming a confidence level of 0.95. In each hospital, five respondents took part in the survey. The first cohort of respondents named ‘Directors’ consisted of hospital directors or employees authorized by them, mostly HR specialists. The other four groups were: ‘Hybrid Doctors Managers’ (individuals who had the roles of both doctor and manager); ‘Hybrid Nurses Managers’ (having the roles of both manager and nurse); ‘Physicians’; ‘Nurses’. Due to the ordinal nature of the data, the chi-square test of independence was used and the V-Cramer coefficient was determined. To indicate significant discrepancies between the responses of the respondents’ cohorts, the Kruskal–Wallis rank test was conducted.

**Results and discussion:**

Various groups of respondents perceive hospital attractiveness as an employer differently. While the opinions of medical personnel are more or less homogeneous, the cohort of employees responsible for HRM are less consistent with regard to their perception of hospital attractiveness. Additionally, ‘Directors’ highlight tangible factors determining hospital desirability. Moreover, their hierarchy of the top five factors influencing EB clearly exposes their quantitative orientation towards hospital performance management. Medical personnel hierarchies of the determinants expose qualitative orientation. Excluding country-dependent factors, such as regularity of remuneration payment, the professionals value such determinants as a nice work atmosphere, cooperation with colleagues (specialists), good working conditions and, most importantly, employment stability. The last determinant results from generational and gender tendencies (feminization), and yet it stands in contradiction with a tendency of flexible employment implemented in most developed countries due to a lack of medical personnel.

**Conclusions:**

The results showed the importance of adapting employer branding strategies to the medical professional groups (doctors and nurses). This is because their perception of employers’ attractiveness differ. In addition, elements of the profession genotypes play an important role in how the physicians and nurses value various factors creating the employers’ attractiveness. The research also revealed the fact that top managers or HR specialists can wrongly identify the hospitals attractiveness since they are more quantitatively than qualitatively oriented. For this reason, they may implement inefficient EB strategies.

## Background

Human resources (HR) along with finances and infrastructure create the fundaments of healthcare systems [1]. The challenges arising from a shortage of financial resources and a need for improvements to infrastructure have been discussed in literature for decades now. By contrast, the issue of HR deficiency has only attracted the attention of researchers fairly recently. This is mainly because of an increased demand for healthcare services caused not only by aging societies [2] but also by an augmentation of civilization diseases [3]. Most developed and underdeveloped countries struggle to deliver the required number and quality of healthcare procedures, mainly due to a shortage of doctors and nurses. Analysis of the World Health Organization's Global Health Workforce Statistics reveals, however, that some countries struggle more from this shortage than others [4]. Generally, developed countries continue to attract and retain medical personnel by creating interesting job opportunities [5] and supporting the inflow of key medical personnel through intentional migration policy [6]. By contrast, underdeveloped countries experience severe problems in retaining medical personnel [7–9] and the major effect of this is intensifying emigration. Interestingly, Poland, which is perceived as a developed country nowadays with its GDP for 2019 reaching 4.34% [10] and GDP per capita in the same year amounting to 31 393 USD [11], experiences challenges that are comparable to those of underdeveloped counties, namely an evident deficiency of physicians and nurses. In addition, Poland was ranked the lowest in the EU in 2017 with 23.79 physicians per 10 000 inhabitants [12]. Furthermore, there are 5.1 nurses per 1 000 inhabitants in the country, which also positions Poland among the lowest in the EU [13]. The most crucial antecedents of doctors and nurses deficiency in Poland are:

- The inefficiency of the education system
  Poland educates too few doctors, and their development, resulting mainly in the achievement of the status of specialists (independent doctors), is not only bureaucratic, it is time-consuming and also less related to practice (direct contact with patients) than in other EU countries. In the case of the nursing profession, the number of places in higher education seems to be satisfactory, but due to the decline in the attractiveness of this profession, the size of the cohorts of candidates for these studies is systematically decreasing.
- Migration of medical personnel
  The size of migrating medical personnel cohorts outside Poland is unknown as no current statistics are kept. Estimated values ​​are determined either on the basis of reports of organizations associating both professions or on the basis of research on the tendency of the personnel to migrate. Therefore, by 2015, the Supreme Chamber of Physicians and Dentists issued as many as 9 288 certificates allowing physicians to practice abroad, especially within the EU. This number constitutes 7.2% of the population of doctors [14]. In the period from 2008 to 2013, the Supreme Chamber of Nurses and Midwives issued a total of 6 799 such the certificates [15]. One of the main reasons for emigration is low salary [16]. Both groups of professionals complain about development opportunities, a lack of professional perspectives and the poor management competences of their supervisors [17, 18]. Due to unsatisfactory salaries, doctors take up jobs in many places and work excessively. In addition, research conducted on a group of 318 doctors revealed that 52% of the studied population worked in 2–3 healthcare facilities, 21% in 4–5, and 4% in more than 5 healthcare facilities. Of the respondents, 10% admitted to working 2–3 times longer than a full-time equivalent (FTE) role, which is more than 10 hours a day, including weekends. Among doctors, 2% declare working 18 hours each day. As a result of the above pathologies, they experience occupational burnout syndrome [19].

The increasing shortage of HR in the Polish healthcare system makes the recruitment and retention of the medical personnel a key challenge for healthcare providers, particularly hospitals, which are people-driven organizations. Therefore, hospitals in particular should constantly improve HRM practices; however, this is not what happens in reality. Hospitals focus more on personnel administration than the improvement of HRM practices aimed at the acquisition and retention of valuable HR [20]. In this context, a visible need for adequate HRM actions taken by strategic healthcare providers can be identified. However, due to the complexity of possible HRM interventions which can be performed, hospitals could first concentrate on the development of an employer brand. This is because this HRM practice results not only in the more effective recruitment of targeted candidates but also increases the HR retention potential of organizations [21].

Employer branding (EB) is not a new concept. Its roots date back to the nineteen-nineties, when the first paper was published. The paper not only defined the concept but also highlighted its importance. The authors of the concept defined employer branding as “the package of functional, economic and psychological benefits provided by employment, and identified with the employing company” [22]. At present, this first definition of EB is regarded as neutral and general [23]. Today's definitions clearly emphasize the importance of target groups to which information about the tangible and intangible advantages of the employer is directed and which distinguish it from other employers [24]. The literature provides a set of advantages of a strong employer image, such as: maintenance of employees’ commitment [23], the ability to attract and retain talented employees [25] and higher engagement [26]. Moreover, the analysis of papers focused on EB drivers shows that the image of an employer of choice is determined by attributes that have long been associated with organizational attractiveness, such as remuneration, social capital and development opportunities [27].

With regard to the healthcare labor market in Poland, there is a visible need for hospitals to build an employer brand so their potential to attract and retain key medical personnel could increase. However, taking into account a modern approach to the EB process, this can be done only when segmentation of the employees’ population is performed. Such an approach calls for a study penetrating the researched reality from various perspectives, especially from the point of view of physicians and nurses. To our knowledge, no study investigating attributes of hospitals as attractive employers has been done either in Poland or in other countries. Moreover, in contrast to our study, research projects that have already been performed were single-dimensional, whereas ours is multidimensional.

## Objective

The objective of this study is to assess the importance of various factors influencing medical personnel’s perception of a hospital’s attractiveness as an employer. Specifically drawing from a contextual approach [28], the study aims not only to identify the importance of certain factors from various perspectives but most of all, to detect differences in the hierarchies of the determinant bundles among various groups of respondents. Additionally, the perceptions of five different perspectives of internal stakeholders are investigated. These stakeholders are: ‘Directors’ (top managers alias HR professionals within hospitals or hospitals’ employees), hybrid line managers (physicians and nurses performing managerial roles), ‘Doctors’ and ‘Nurses’.

## Methodology

Data required for attaining the study aims was gathered from research conducted in 285 Polish hospitals, assuming a confidence level of 0.95. A simple draw was carried out which enabled the selection of hospitals for the study. In the absence of consent to conduct a study in a given facility, another unit from the survey frame was drawn, excluding the units previously drawn. The adopted methodology for selecting hospitals for the research enabled the research to be considered as being representative of the population of hospitals in Poland. The survey primarily targeted hospital directors due to the fact that in practice, these managers are still to a large extend involved in HRM activities in hospitals. There were also four other groups of respondents participating in the study. The multi-perspective research design was deemed important because of the possible overstatement of answers known as officialization and the nested professional features of physicians and nurses. In addition, the first group (‘Directors’) consisted of various respondents accountable for human resource management. In addition, underdevelopment of HRM activities in Polish hospitals often causes there to be neither a manager or specialist nor an HR department per se focusing on HRM. In medical entities, there are departments which focus on personnel administration. As a result, all managerial responsibilities are in the hands of general managers (directors). Taking this into consideration, the outsourced research company contacted directors first. Directors familiarized themselves with the thematic scope of the survey and made decisions regarding the personal response or delegation of this activity. Most commonly, they highlighted subordinates who were responsible for HRM. The group of respondents consisted of: 57 directors (20%), 138 HR managers (48%), 83 HR specialists (29%) and 7 other respondents (2%), e.g. PR specialists. In addition, due to diversity of the group, the Kruscal-Wallis test was run and its results did not reveal significant dissimilarities among the respondents within the particular answer categories / variables (p > 0.05). Hospitals were asked to forward the survey to other groups of respondents, such as physicians and nurses serving as ward managers and line managers (also named hybrid line managers), physicians and nurses. The perspectives of the ‘Hybrid doctors managers’ (HDMs) and ‘Hybrid nurses managers’ (HNMs) were extremely important due to the fact that they combine both roles, managerial and professional [29]. Because of this, their judgment of the reality differs not only from that of the hospitals’ management but also from physicians and nurses who do not play managerial roles yet manage the medical personnel on a daily basis. There was a total of 285 respondents in each of the five groups (Table 1).

The survey was a part of a larger project entitled *Human Capital Management in Hospitals*. It was designed on the basis of an extensive literature review and discussions among researchers concerning the results of the qualitative stage of the research project which was performed before the survey (multiple case studies were performed within the hospitals). The reliability of the content of the questionnaires were tested with Cronbach’s Alpha coefficient test [30–32]. The results for the particular groups of respondents are as follows: ‘Directors’ 0.827, ‘HDMs’ 0.798, ‘HNMs’ 0.967, ‘Physicians’ 0.776, ‘Nurses’ 0.723. The variances in the results of the Cronbach’s Alpha coefficient test occurred due to the fact that the questionnaires administered to the five groups of the respondents were partially different. They were adjusted to the specifics of the particular group of respondents, thus creating an individual theoretical construct for every group.

This paper was elaborated on the basis of the part of the questionnaires which were aimed at the identification of a set of factors determining the attractiveness of the hospitals as employers (see Variables 1–18 in Table 2). This part was the same in each of the questionnaires. Respondents in each group were asked to appraise the determinants of hospital attractiveness as an employer on a 5-point Likert scale. The reliability of the part of the questionnaires was not checked individually because it does not create an independent theoretical construct and so the results would not be authoritative.

**Table 1:** Characteristics of the researched populations (%)

	Directors	HDMs	HNMs	Physicians	Nurses	Total
Age						
Left blank	7.7	0	0	0	0	1.5
25–34	1.4	0	4.2	0.4	1.8	1.5
35–44	26.3	16.8	33.3	45.3	46.0	33.5
45–54	38.9	43.9	35.8	50.2	49.5	43.6
55–64	24.9	38.2	26.7	3.9	2.8	19.3
65–74	0.7	1.1	0.0	0.4	0.0	0.4
Gender						
Female	80.4	42.5	100	57.2	100	76
Male	19.6	57.7	0	42.8	0	24
Tenure						
Left blank	1.4%	0.0%	0.0%	0.0%	0.0%	0.3%
0–9	23.9%	4.2%	14.0%	26.7%	30.9%	19.9%
10–19	29.8%	32.3%	28.4%	51.2%	42.5%	36.8%
20–29	24.9%	45.3%	41.1%	21.4%	25.6%	31.6%
30–39	16.5%	16.5%	15.8%	0.7%	1.1%	10.1%
40–49	3.2%	1.1%	0.7%	0.0%	0.0%	1.0%
50–59	0.4%	0.7%	0.0%	0.0%	0.0%	0.2%
Educational level						
Secondary	6.7	0	0	0	7.4	2.8
Bachelor degree	0	0	0	0	9.8	2.0
Master degree	82.1	88.8	100	96.8	82.8	90.1
PhD	9.8	6.3	0	3.2	0.0	3.9
Other	1.4	5	0	0	0.0	1.3

Source: Self-elaboration

**Table 2:** Standard deviations (SD) and coefficients of variation (V) expressed in % for the respondents’ cohorts

Variable	Directors		HDMs		HNMs		Physicians		Nurses	
	SD	V[%]	SD	V[%]	SD	V[%]	SD	V[%]	SD	V[%]
1. Attractive salary	0.49	11.4	0.69	16.6	0.76	18.4	0.89	21.8	0.81	19.5
2. The opportunity to develop knowledge and skills	0.44	10.4	0.60	13.9	0.59	13.6	0.65	15.3	0.66	15.5
3. Possibility of implementing innovative medical procedures	0.61	14.8	0.60	14.6	0.66	16.0	0.75	18.3	0.71	17.2
4. Substantive authority of the immediate superior	0.59	13.9	0.72	17.0	0.66	15.5	0.73	17.3	0.66	15.2
5. Good working conditions (equipment, medical equipment)	0.56	12.7	0.53	12.0	0.59	13.2	0.69	15.6	0.64	14.6
6. Good working conditions (infrastructure)	0.55	12.7	0.59	13.6	0.55	12.5	0.83	19.6	0.75	17.1
7. Positive opinion about the hospital as an employer	0.59	13.7	0.67	15.7	0.63	14.5	0.78	18.4	0.83	19.7
8. Distance to academic centers (these located close by)	0.90	21.9	1.03	27.3	1.06	28.0	0.94	23.6	1.01	25.2
9. Positive opinion about the top management	0.67	15.7	0.70	16.7	0.80	19.1	0.93	22.4	0.78	18.2
10. Possibility of flexible working hours	0.62	14.6	0.74	17.9	0.70	16.8	1.03	25.0	0.96	23.1
11. Possibility of providing work based on a flexible form of employment (e.g. contract)	0.59	13.5	0.78	18.7	0.75	18.2	1.00	24.1	1.03	25.4
12. Possibility of continuing outpatient or private treatment at the hospital	0.71	17.3	0.73	18.0	0.74	17.8	0.93	22.9	0.94	23.4
13. Distance from home (hospital located nearby home)	0.71	16.9	0.98	24.7	0.76	18.1	0.80	18.3	0.82	18.9
14. Regular payment of remuneration (on time)	0.59	13.6	0.57	12.8	0.55	12.1	0.52	11.5	0.53	11.7
15. Opportunity to work with specialists	0.54	12.3	0.58	13.3	0.59	13.5	0.55	12.2	0.54	12.1
16. Employment security (stability)	0.56	12.8	0.58	13.0	0.58	13.0	0.57	12.7	0.54	12.0
17. Good relationships with colleagues	0.59	13.4	0.58	13.4	0.56	12.6	0.64	14.1	0.58	12.8
18. Nice work atmosphere	0.61	14.2	0.66	15.3	0.64	14.2	0.63	13.9	0.54	11.9

Data was entered and analyzed using the Statistica 13 software application. With regard to the metric questions, distribution analysis was applied. The structure of the question relating to the issue of hospital attractiveness as an employer made it possible to analyze the relationship between the attractiveness assessment and the position. Due to the ordinal nature of the data, a chi-square test of independence was used and the V-Cramer coefficient was determined. In order to illustrate how the differences in the perception of attractiveness were perceived, a chart was prepared presenting a comparison of the average answers of particular issues for the studied groups. In order to additionally indicate significant discrepancies between the responses of the different groups, a Kruskal–Wallis rank test was performed. When the test showed significant differences (at the significance level of p < 0.05), a multiple comparison of mean ranks was performed.

## Results

### Characteristics of samples

The largest cohort of respondents was within the 45–54 years age range. Importantly, in the case of ‘Hybrid doctors managers’ it was 43.9% of the respondents, for ‘Hybrid nurses managers’ 35.8%, for ‘Doctors’ 50.2% and for ‘Nurses’ 49.5%. Across all respondents, the size of the cohort aged 45–54 was 43.6%. The second largest group of respondents was aged 35–44 (33.5%). In total, both groups accounted for 77.1% of respondents. The age distribution of the respondents is determined primarily by the age of medical staff, doctors and nurses occupying both managerial and operational positions. Women constituted 76% of the respondents. Respondents were also asked about seniority and education. The fact that few respondents declare secondary education is due to the following reasons: first, the ‘Directors’ group could include people authorized by directors to answer interviewers, e.g. employees of the personnel management department dealing with administrative matters. Secondly, in the group of ‘Nurses’, there are still those who entered the labor market in a situation where higher education was not required and they are still active on the labor market. In addition, the regulations concerning educational requirements for nurses were implemented in 2011.

## Determinants of hospital attractiveness—multidimensional perspective

The in-depth analysis of the literature reveals that the specificity of the cohorts of respondents participating in the study is diverse [28]. They can also vary internally and because of this, the data analysis began with the calculation of standard deviations (SD) and coefficients of variation (V). The results included in Table 2 show that the answers of the respondents are consistent. There are only few deviations higher than 20% within the respondents groups; however, they do not exceed 30%. Interestingly, the largest number of variations can be noticed with first physicians, and then nurses.

Further analysis of the data focused on examining the relationship between the type of cohort (position) and the factors determining the attractiveness of the hospital as an employer (Table 3). The chi-square statistic and the V-Cramer coefficient were selected for the analysis of the relationship. The analysis revealed a significant relationship between the individual factors of hospital attractiveness and the type of cohort in the vast majority of the studied factors. Only in the case of two variables was no such relationship identified, namely 'good working conditions (equipment, medical equipment)' and 'positive opinion about the hospital as an employer'. The strongest relationship between the studied variables was identified in relation to the variables: 'nice work atmosphere' (0.1436), 'possibility of providing work based on a flexible form of employment, e.g. contract' (0.1387) and 'the opportunity to develop knowledge and skills' (0.1268). The least correlated are: 'good working conditions (equipment, medical equipment)' (0.0656), 'positive opinion about the hospital as an employer' (0.0658) and 'regular payment of remuneration (on time)' (0.0774). It was decided to test the similarities and the differences in the assessment of the importance of individual attractiveness factors using the mean values.

**Table 3:** The relationship between the responses for individual variables and respondents’ cohorts

Variable	Chi-square statistics	df	p	V-Cramer factor
1. Attractive salary	89.5300	16	**0.0000**	0.1253
2. The opportunity to develop knowledge and skills	68.7039	12	**0.0000**	0.1268
3. Possibility of implementing innovative medical procedures	37.7770	16	**0.0016**	0.0814
4. Substantive authority of the immediate superior	34.2841	16	**0.0050**	0.0776
5. Good working conditions (equipment, medical equipment)	24.5639	16	0.0779	0.0656
6. Good working conditions (infrastructure)	80.8513	16	**0.0000**	0.1191
7. Positive opinion about the hospital as an employer	24.6536	16	0.0762	0.0658
8. Distance to academic centers (these located close by)	70.7004	16	**0.0000**	0.1114
9. Positive opinion about the top management	52.6908	16	**0.0000**	0.0962
10. Possibility of flexible working hours	70.6230	16	**0.0000**	0.1113
11. Possibility of providing work based on a flexible form of employment (e.g. contract)	109.6231	16	**0.0000**	0.1387
12. Possibility of continuing outpatient or private treatment at the hospital	71.7150	16	**0.0000**	0.1122
13. Distance from home (hospital located nearby home)	79.5083	16	**0.0000**	0.1181
14. Regular payment of remuneration (on time)	25.6291	12	**0.0121**	0.0774
15. Opportunity to work with specialists	28.392	8	**0.0004**	0.0998
16. Employment security (stability)	39.4856	12	**0.0001**	0.0961
17. Good relationships with colleagues	44.7397	12	**0.0001**	0.1023
18. Nice work atmosphere	88.1952	12	**0.0000**	0.1436

Source: Self-elaboration

**Fig. 1:**
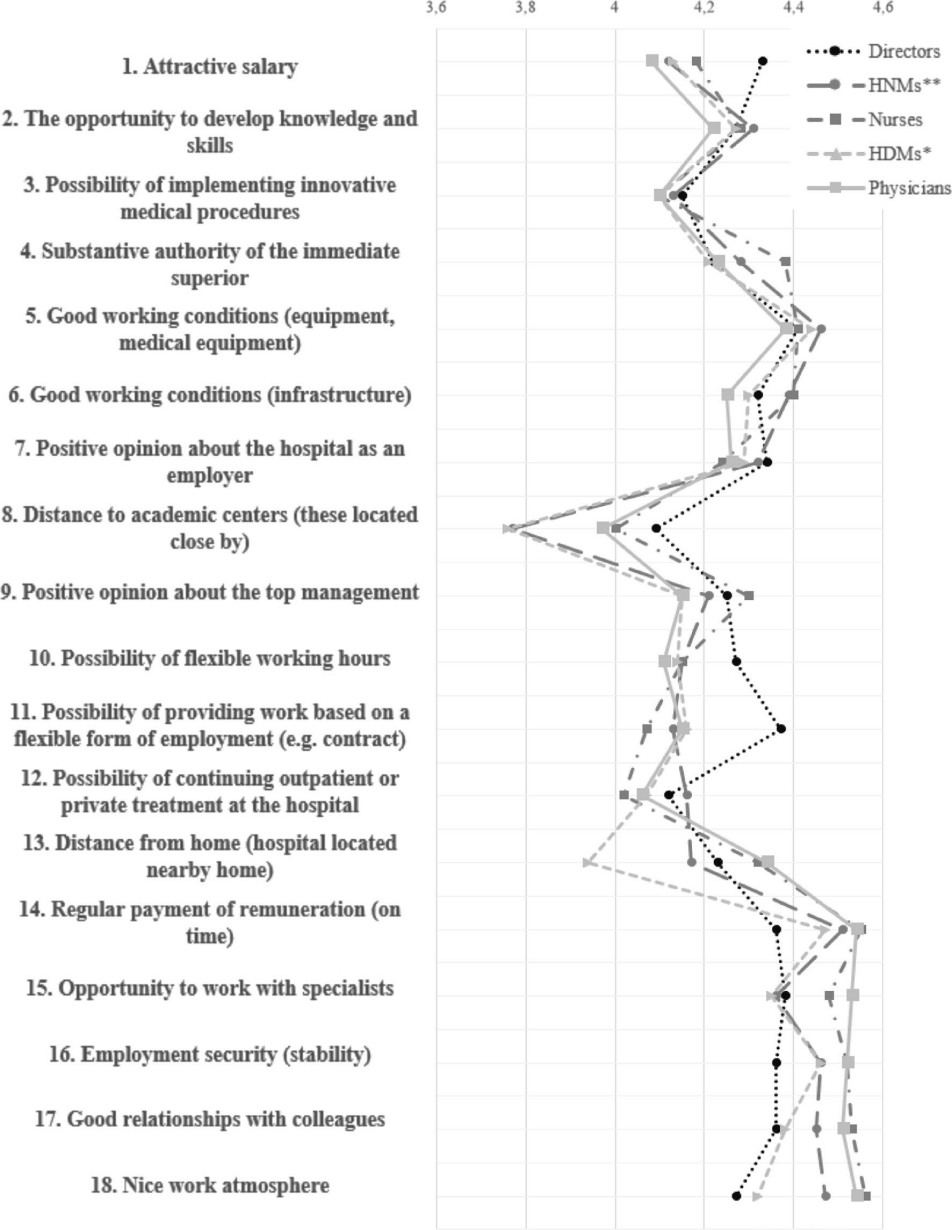
Mean values for variables in each cohort of respondents ( Source: Self-elaboration)

The calculation of means for each cohort demonstrated that sets of the most and least important variables determining hospitals’ attractiveness as employers vary (Fig. 1). Respondents belonging to the ‘Directors’ cohort highly valued ‘good working conditions (equipment, medical equipment)’ (4.41), ‘opportunity to work with specialists’ (4.38), and ‘possibility of providing work based on a flexible form of employment (e.g. contract)’ (4.37). ‘HDMs’ highly valued ‘regular payment of remuneration (on time)’ (4.47), ‘employment security’ (4.46), and ‘good working conditions (equipment, medical equipment)’. ‘HNMs’ assigned the highest values to ‘regular payment of remuneration (on time)’ (4.51), ‘nice work atmosphere’ (4.47) and ‘good working conditions (equipment, medical equipment)’ (4.46). For ‘Physicians’ the most important features of a good employer were ‘regular payment of remuneration (on time)’ (4.54), ‘nice work atmosphere’ (4.54) and ‘opportunity to work with specialists’ (4.54). Partly similar answers were given by ‘Nurses’; for them, the most important factors of a hospital as the most important features of good employer were: ‘nice work atmosphere’ (4.56), ‘regular payment of remuneration (on time)’ (4.55) and ‘good relationships with colleagues’ (4.53).

The existence of statistically significant relationships between the groups of respondents, the factors influencing the attractiveness of the hospital (Table 3) and the determination of differences in the hierarchy of importance of individual factors for individual cohorts participating in the study (Fig. 1) prompted the analysis of differences in the respondents' answers. Significant differences in the distribution of responses were found for several variables. The variables most differentiating the opinions of individual professional groups in the studied population were (Table 4):

- ‘Distance from home (hospital located nearby home)’, ‘good relationships with colleagues’ and ‘nice work atmosphere’ where significant differences in the distribution of the answers of four groups of respondents were identified;
- ‘Opportunity to work with specialists’ where significant differences in the distribution of answers of three groups of respondents were identified;
- ‘Attractive salary’, ‘distance to academic centers (these located close by)’, ‘regular payment of remuneration (on time)’, ‘employment security (stability)’ where significant differences in the distribution of answers of two groups of respondents were identified.

**Table 4:** Distribution of answers of the researched cohorts

Variable	Kruskal–Wallis statistics	p*	Significant differences in the distributions between the cohorts
1. Attractive salary	14.453	**0.006**	‘Directors’—‘HDMs’ *‘Directors’—‘HNMs’*
2. The opportunity to develop knowledge and skills	3.965	0.411	−
3. Possibility of implementing innovative medical procedures	1.182	0.881	−
4. Substantive authority of the immediate superior	13.714	**0.008**	‘Directors’—‘Nurses’
5. Good working conditions (equipment, medical equipment)	1.603	0.808	−
6. Good working conditions (infrastructure)	12.465	**0.014**	*‘HDMs’—‘Nurses’*
7. Positive opinion about the hospital as an employer	0.440	0.979	−
8. Distance to academic centers (those located close by)	23.289	**0.000**	‘Directors’—‘HDMs’ ‘Directors’—‘HNMs’ *‘HNMs’—‘Nurses’*
9. Positive opinion about the top management	10.166	**0.038**	‘HDMs’—‘Nurses’
10. Possibility of flexible working hours	5.723	0.221	−
11. Possibility of providing work based on a flexible form of employment (e.g. contract)	14.755	**0.005**	‘Directors’—‘Physicians’
12. Possibility of continuing outpatient or private treatment at the hospital	2.639	0.620	−
13. Distance from home (hospital located nearby home)	42.449	**0.000**	‘Directors’—‘HDMs’ ‘HDMs’—‘Physicians’ ‘HNMs’—‘Physicians’ ‘HDMs’—‘Nurses’ *‘HNMs’—‘Nurses’*
14. Regular payment of remuneration (on time)	17.753	**0.001**	‘Directors’—‘Physicians’ ‘Directors’—‘Nurses’ *‘Directors’—‘HNMs’*
15. Opportunity to work with specialists	23.480	**0.000**	‘Directors’—‘Physicians’ ‘HDMs’—‘Physicians’ ‘HNMs’—‘Physicians’
16. Employment security (stability)	16.147	**0.003**	‘Directors’—‘Physicians’ ‘Directors’—‘Nurses’
17. Good relationships with colleagues	24.512	**0.000**	‘Directors’—‘Physicians’ ‘Directors’—‘Nurses’ ‘HDMs’—‘Physicians’ ‘HDMs’—‘Nurses’
18. Nice work atmosphere	57.157	**0.000**	‘Directors’—‘HNMs’ ‘Directors’—‘Physicians’ ‘Directors’—‘Nurses’ ‘HDMs’—‘Physicians’ *‘HDMs’—‘HNMs’*

Source: Self-elaboration

* Distributions that were not lower than 0.05 but were close to this limit; lower than 0.10 are marked in italics

The cohorts who differentiated themselves the most were:

- ‘Directors’ vs ‘Physicians’ (6 times),
- ‘Directors’ vs ‘Nurses’ (5 times),
- ‘Directors’ vs ‘HNMs’ (4 times),
- ‘HDMs' vs ‘Physicians’ (4 times),
- ‘HDMs’ vs ‘Nurses’ (4 times).

## Discussion

There has been a debate in the literature about the effective management of HR in healthcare entities. This is due to the fact that the resource is finite. Researchers approach the area of investigation not only from different perspectives (micro-, mezzo-, macro-) but also focusing on diverse issues, such as migration [17], retention [33] and motivation [34]. Although a significant number of studies have deepened knowledge regarding the management of HR in healthcare, none of them have focused precisely on employer branding. Furthermore, a limited number of them have approached the researched reality from distinct points of view. The study aimed at the identification of key determinates of the hospitals’ brand. With the assumption that the cohorts of respondents differ, the research intended to identify variances in the hierarchies of the determinants. Both of the main premises of the study were proven. The results of the analysis not only displayed key factors determining the hospitals’ attractiveness as employers but first and foremost identified the sets of factors defined by the cohorts as being strategic for the employers’ desirability [Table 5].

**Table 5:** Five most important features of hospital attractiveness for each of the researched cohorts

Directors	HDMs	HNMs	Physicians	Nurses
5. Good working conditions (equipment, medical equipment)	14. Regular payment of remuneration (on time)	14. Regular payment of remuneration (on time)	14. Regular payment of remuneration (on time)	*18. Nice work atmosphere*
15. Opportunity to work with specialists	16. Employment security (stability)	*18. Nice work atmosphere*	*18. Nice work atmosphere*	14. Regular payment of remuneration (on time)
11. Possibility of providing work based on a flexible form of employment (e.g. contract)	5. Good working conditions (equipment, medical equipment)	5. Good working conditions (equipment, medical equipment)	15. Opportunity to work with specialists	**17. Good relationships with colleagues**
14. Regular payment of remuneration (on time)	**17. Good relationships with colleagues**	16. Employment security (stability)	16. Employment security (stability)	16. Employment security (stability)
16. Employment security (stability)	15. Opportunity to work with specialists	**17. Good relationships with colleagues**	**17. Good relationships with colleagues**	15. Opportunity to work with specialists

Source: Self-elaboration

*Numbers from this table assigned to the determinants were maintained in order to facilitate analysis

Considering the data included in Table 4, it can be identified that variations are evident between ‘Directors’ and the other groups of respondents. In addition, ‘Directors’ gave the highest appraisal to ‘Good working conditions (equipment, medical equipment)’. This factor was appraised relatively highly by ‘HDMs’ and ‘HNMs’. However, it was not considered as the most important factor of the hospitals’ attractiveness by ‘Physicians’ and ‘Nurses’. The results of the study partly confirm the findings of research performed on doctors, who highlighted work conditions (i.e. infrastructure) of healthcare entities located in rural settings as being crucial for their work satisfaction [35]. In addition, the hybrid managers (HM) highlighted this factor. On contrary ‘Physicians’ and ‘Nurses’ did not find the determinant to be highly important. This variation with regard to perceived importance might be caused by differences in the work obligations spectrum. HM are obligated to deliver resources [36], both intangible and tangible. Because of this, the aspect becomes more important for managers than for the individual users. The perception of this factor as the most important by the management, especially ‘Directors’, may also be due to the fact that the equipment infrastructure in Polish hospitals is aging. At the same time many hospitals cannot afford to buy new equipment due to very limited financial resources. Simultaneously managers, both hybrid and non-hybrid, realize that modern equipment can improve the quality and efficiency of medical services provided.

In the hierarchy of determinant importance, ‘Directors’ indicated ‘Opportunity to work with specialists’ as being second. This factor was appraised as relatively low by ‘HDMs’. However, it was appraised so high that it made third place among the factors among ‘Physicians’. Furthermore, it was perceive as important by ‘Nurses’ as it made the fifth place in the ranking in Table 5. The high level of assessment of this factor by ‘Directors’ may be caused by the fact that they are counting on the appearance of intra-organizational learning mechanisms. They may also be familiar with the specifics of the medical profession and the traditions associated with it. In addition, learning from older colleagues and consulting difficult cases are written into physician’s genotype [28]. This characteristic is in fact confirmed by the results of the study. ‘Physicians’ appraised the determinant higher than their direct managers—‘HDMs’. This historical conditioning does not apply to nurses; however, when entering the healthcare system or at the beginning of their career, they also need support from their more experienced colleagues [37].

‘Directors’ appraised ‘Possibility of providing work based on a flexible form of employment (e.g. contract)’ as an important determinant of hospitals attractiveness. This factor, however, was not found to be an important determinant from the perspectives of the other respondents. There is a set of rationales for this difference. Additionally, both of the professions are feminized. Nursing is feminized by tradition, while the profession of physicians has been recently becoming significantly more feminized than in the past. The feminization of the physicians’ profession results in higher desirability for stable employment [28]. This can be confirmed by the fact that factors associated with stability of employment such as ‘Regular payment of remuneration (on time)’ and ‘Employment security (stability)’ were valued highly by the medical personnel cohorts.

The other rationale for such results is that flexible forms of employment give ‘Directors’ more flexibility concerning the timeframes and the amount of work they delegate to contactors (for most self-employed physicians, Polish Labor Law clearly regulates the working time of medical personnel). However, with such a large shortage of doctors, these provisions are often circumvented. Top management is encouraged to set up a business and provide services through it, while resigning from employment under an employment contract. This solution is favorable for the physicians due to an opportunity to increase their income, but at the same time, it evidently limits their possibilities to develop knowledge and skills. This is due to the fact that they have very limited time to attend training activities. Additionally, Polish doctors break records when it comes to hours worked per day, week, month or year. The research conducted on a group of 624 doctors shows that 59% of them worked 24 h without a break at least once a year [38]. As the analysis of our research shows, this negative tendency may self-regulate over time. While the management may continue to offer flexible employment, medical personnel may not agree to the solution. This is due to the fact that the personnel appreciate stability more often. This quest for stability will be strengthened for two reasons. Firstly, Polish society is clearly aging, and secondly, younger generations of doctors do not want to expose themselves to the risks posed by overtime work. Young doctors want to balance their work with personal life, just like any typical representative of the Y or Z generation [39].

The forth determinant identified as being important in the context of employer branding in the opinion of ‘Directors’ is ‘Regular payment of remuneration (on time)’. The relatively high weight of this factor ascribed by this group of respondents may result from another challenge faced by hospitals managers in Poland. On the one hand, managers are encouraged to provide medical services to meet the health needs of citizens. On the other hand, they are aware of the limitations in the volume of services contracted by the National Health Fund. They know that exceedance of a contract with the public payer may or may not result in covering the costs of the delivered services. As a result, the management of public sector service providers is accompanied by uncertainty that may result in the fear of late payment of employees' wages. It should be noted that this concern is not only due to concern over the well-being of employees, but also the desire to maintain good relations with trade unions, which in the Polish health system are very active and often significantly hinder the functioning of healthcare entities.

In the case of this determinant, the opinions of other groups of respondents differ. ‘HDMs’, ‘HNMs’ and ‘Physicians’ perceive this factor as the most important. The declarations of ‘Nurses’ positioned this factor in second place. As mentioned earlier, the high value assigned by the medical personnel to this factor results from the need for employment stability. Moreover, in the case of ‘Physicians’, the result contradicts a study which exhibited that the professionals succumb to *homo economicus* syndrome [40]. This finding is supported by the fact that ‘attractive salary’ was not within the top five determinants of the hospitals’ attractiveness. ‘Nurses’ appraised ‘Nice atmosphere’ higher than ‘Regular payment of remuneration’. This result of the study confirmed the findings of research by Petrides and McManus 2004 [41]. The researchers detected that candidates who enter studies aimed at the nursing profession are more social than research oriented. The hierarchization exhibits a basic feature of nursing, namely the creation of a good atmosphere for more effective patient recovery. There might be another reason for ‘Nurses’ to highlight ‘nice atmosphere’ as being important. The term is often associated with a good relationship with coworkers or, in other words, positive social capital. It is a ‘natural weapon’ for the professionals to defend themselves against emotional exhaustion and because of this, nurses can value this aspect highly. In addition, the study performed by Kowalski et al. confirmed that “efforts to create a participative working atmosphere, with readiness to provide mutual support and to share values and objectives in a hospital and to reduce workload may minimize the risk of burnout in the form of emotional exhaustion in hospital nurses” [42, p. 1661].

‘Nice work atmosphere’ is not included in the top five determinants of hospital attractiveness by ‘Directors’. However, ‘Physicians’ also value the factor highly. This finding confirms the conclusions of Wang et al. [35], who discovered the strategic importance of work atmosphere for physicians. According to their study, a nice work atmosphere leads to the higher retention of doctors. Moreover, it can significantly reduce physicians' burnout, especially the emotional exhaustion score and the depersonalization score and as a result, can increase Physician Input [28]. There was another intangible factor which did not make the Directors’ top five determinants of EB but can be found in the top five of the other cohorts of respondents, namely ‘Good relationships with colleagues’. The exclusion of these factors from the most important determinants of EB in the opinion of ‘Directors’ confirms the findings of the orientation of Polish hospitals, which revealed that they are focused on costs rather than human capital [20]. Moreover, taking into consideration the fact that some of the respondents from the cohort have a medical educational background, the finding exposes a managerial competences gap. In addition, multiple studies found that HDMs lack managerial competences and yet there are unwilling to develop them [43, 44]. This conclusion is also supported by our study. All cohorts of medical personnel defined this factor as being strategically important. Such a result confirms the respondents’ awareness of the strategic importance of social capital (therapeutic team) in the performance of medical procedures. Furthermore, the fact that the respondents from medical personnel perceive ‘Good relationships with colleagues’ as being important, this once again at least partially confirms the results of the study by Kowalski et al. 2010 and also our earlier observations. In addition, nurses value this determinant considerably higher than other medical cohorts of respondents [42].

‘Employment security (stability)’ was appraised by ‘Directors’ as also being strategically important for medical personnel. However, the factor was hierarchized as fifth. Such a finding does not stand in contradiction to those considering flexibility of employment. In fact, this confirms an awareness of at least some of the needs of medical personnel in hospitals. Nevertheless, the finding once more confirms a stronger orientation towards cost than towards human capital and as a consequence, this has an impact upon quality.

## Conclusion

There is an evident shortage of medical personnel in Poland and yet a scarce number of hospitals improve their HRM practices in order to attract and retain employees, especially considering that other developed countries lure professionals to emigrate and commence their careers there. The improvement of HRM practices becomes the essential condition not only for medical personnel attraction in working for the healthcare entities but also for the improvement of professional performance. This relationship is evidently forgotten in Poland. Thus, problems arising from inefficient medical personnel management are being uncovered under the SARS-CoV-2 pandemic. Additionally, the Polish government is able to deliver the necessary infrastructure but there is a visible lack of personnel willingly relocating to permanent or temporary Covid hospitals. The enhancement of HRM practices cannot be performed in the short term and also it should be performed sequentially. Due to this fact, the paper aimed to identify the most important factors determining hospitals’ attractiveness as employers. Moreover, the relationship between the type of respondent and the combination of the factors was analyzed. The major finding of the study is that there is an association between the determinants of EB and the type of respondent cohorts employed within hospitals. The hierarchies differ. These differences are caused either by the respondents perspective and the role performed within the hospital (top management—HMs—single professionals), or by features characterizing the professions (physicians and nurses).

The results of the study sustain a foundation for hospitals’ EB strategy creation. Considering the fact that the strategy should be aimed at the attraction and retention of the most strategic groups of employees within an organisation [45], in the case of the hospitals, physicians and nurses should be targeted. Of course, this does not mean that less consideration should be paid to other employees. It should be assumed that they will benefit from the improvements as a consequence of EB strategy implementation. The elaboration of the EB strategy should be initiated with an employer value proposition (EVP) definition. Considering the results of the study—the characteristics of the key medical personnel populations (i.e. ageing, generational diversity, expectations of generation Y) and the context of the hospitals’ performance—it is reasonable to suggest that the EVP should be derived from sustainable HRM assumptions. In addition, values assimilated with sustainable HRM are, for example, the transfer of knowledge and skills to the future generation (competence development), the recognition of older workers and well-being (social sustainability, reduction of stress factors influence) [46, 47]. Establishing EVP allows the framework of the employer of choice strategy to be defined. Another task, however, is to designate actions that will increase the attractiveness of a given employer to both current employees and job candidates. These activities are primarily related to the improvement of HRM [44]. Referring to the research results, and particularly taking into account the most important factors of the attractiveness of hospitals as employers as indicated by doctors and nurses (see Table 5), it should be stated that the basic areas of HRM that should be improved are:

- Compensation—its regularity;
- HR development—the creation of knowledge-sharing mechanisms and the development of interpersonal competences which support the knowledge development processes;
- Social capital management—organisational culture management aimed at culture oriented towards innovation [48, 49], the improvement of communication systems and the management of teams;
- Changes in the forms of employment—from flexible to more stable.

During the process of employer branding elaboration, it is also necessary to plan activities aimed at effective brand communication, both internally and externally. When considering internal communication, it is extremely important to make sure that the appropriate system is unobstructed. In addition, the inefficiency of the communication system in organisations results in a lack of employees’ knowledge about employer values, and thus in a lack of identification with them. As a consequence, hospitals may lose the ability to use the most effective "tool" for recruiting a new employee, which is a current employee, who in fact acts as a brand ambassador.

In the case of hospitals, awareness of the importance of medical personnel serving as intentional or non-intentional brand ambassadors is particularly important. This is because medical professionals regularly and actively participate in various types of professional meetings, mainly training activities, during which they share their opinions about their employers. Their activity serves as a primary tool for the external communication of the employer brand. Another important tool is social media that reaches representatives of the younger generation. In summarising the recommendations related to the creation of a hospital’s EB strategy, it should be underlined that the strategy should be elaborated by teams consisting of representatives from various groups of employees. Additionally, such a final recommendation comes from the fact that the study revealed that the attractiveness of the hospitals as employers is perceived differently by the internal stakeholders of these organisations. Therefore, not only should HR specialists, marketing specialists and managers be involved in the process, but most of all, individual employees, especially doctors and nurses.

## Data Availability

The datasets used and/or analyzed during the current study are available from the corresponding author upon reasonable request.
